# Role of Cardiac Energetics in Aortic Stenosis Disease Progression: Identifying the High-risk Metabolic Phenotype

**DOI:** 10.1161/CIRCIMAGING.122.014863

**Published:** 2023-10-17

**Authors:** Shveta Monga, Ladislav Valkovič, Saul G. Myerson, Stefan Neubauer, Masliza Mahmod, Oliver J. Rider

**Affiliations:** Division of Cardiovascular Medicine, Radcliffe Department of Medicine, University of Oxford, United Kingdom (S.M., L.V., S.G.M., S.N., M.M., O.J.R.).; Department of Imaging Methods, Institute of Measurement Science, Slovak Academy of Sciences, Bratislava, Slovakia (L.V.).

**Keywords:** aortic valve stenosis, magnetic resonance imaging, metabolism

## Abstract

**Background:**

Severe aortic stenosis (AS) is associated with left ventricular (LV) hypertrophy and cardiac metabolic alterations with evidence of steatosis and impaired myocardial energetics. Despite this common phenotype, there is an unexplained and wide individual heterogeneity in the degree of hypertrophy and progression to myocardial fibrosis and heart failure. We sought to determine whether the cardiac metabolic state may underpin this variability.

**Methods:**

We recruited 74 asymptomatic participants with AS and 13 healthy volunteers. Cardiac energetics were measured using phosphorus spectroscopy to define the myocardial phosphocreatine to adenosine triphosphate ratio. Myocardial lipid content was determined using proton spectroscopy. Cardiac function was assessed by cardiovascular magnetic resonance cine imaging.

**Results:**

Phosphocreatine/adenosine triphosphate was reduced early and significantly across the LV wall thickness quartiles (Q2, 1.50 [1.21–1.71] versus Q1, 1.64 [1.53–1.94]) with a progressive decline with increasing disease severity (Q4, 1.48 [1.18–1.70]; *P*=0.02). Myocardial triglyceride content levels were overall higher in all the quartiles with a significant increase seen across the AV pressure gradient quartiles (Q2, 1.36 [0.86–1.98] versus Q1, 1.03 [0.81–1.56]; *P*=0.034). While all AS groups had evidence of subclinical LV dysfunction with impaired strain parameters, impaired systolic longitudinal strain was related to the degree of energetic impairment (*r*=0.219; *P=*0.03). Phosphocreatine/adenosine triphosphate was not only an independent predictor of LV wall thickness (*r*=−0.20; *P*=0.04) but also strongly associated with myocardial fibrosis (*r*=−0.24; *P*=0.03), suggesting that metabolic changes play a role in disease progression. The metabolic and functional parameters showed comparable results when graded by clinical severity of AS.

**Conclusions:**

A gradient of myocardial energetic deficit and steatosis exists across the spectrum of hypertrophied AS hearts, and these metabolic changes precede irreversible LV remodeling and subclinical dysfunction. As such, cardiac metabolism may play an important and potentially causal role in disease progression.

**CLINICAL PERSPECTIVE**

A gradient of myocardial energetic deficit and steatosis exists across the spectrum of hypertrophied aortic stenosis hearts, and these metabolic changes precede irreversible left ventricular remodeling and subclinical dysfunction. Impaired cardiac energetics occur before a significant increase in left ventricular wall thickness and has a significant association with impairment in myocardial strain and development of myocardial fibrosis, which is likely to be relevant clinically. Modulating myocardial metabolism may prove to be a promising strategy in improving outcomes of patients with aortic stenosis. We are currently testing this intriguing possibility in a clinical trial (NCT05256758) evaluating the effect of metabolic modulation on cardiac physiology in moderate-severe aortic stenosis.

**See Editorial by Sosnovik and Elmariah**

Aortic stenosis (AS) is characterized by both progressive valve obstruction and left ventricular (LV) hypertrophic remodeling (Figure 1). Conventional thinking holds that hypertrophy is a compensatory response to increased workload, serving to minimize wall stress and maintain contractile function. However, several lines of evidence—preclinical and epidemiological—highlight the maladaptive features of chronic hypertrophy.^1,2^

**Figure 1.**
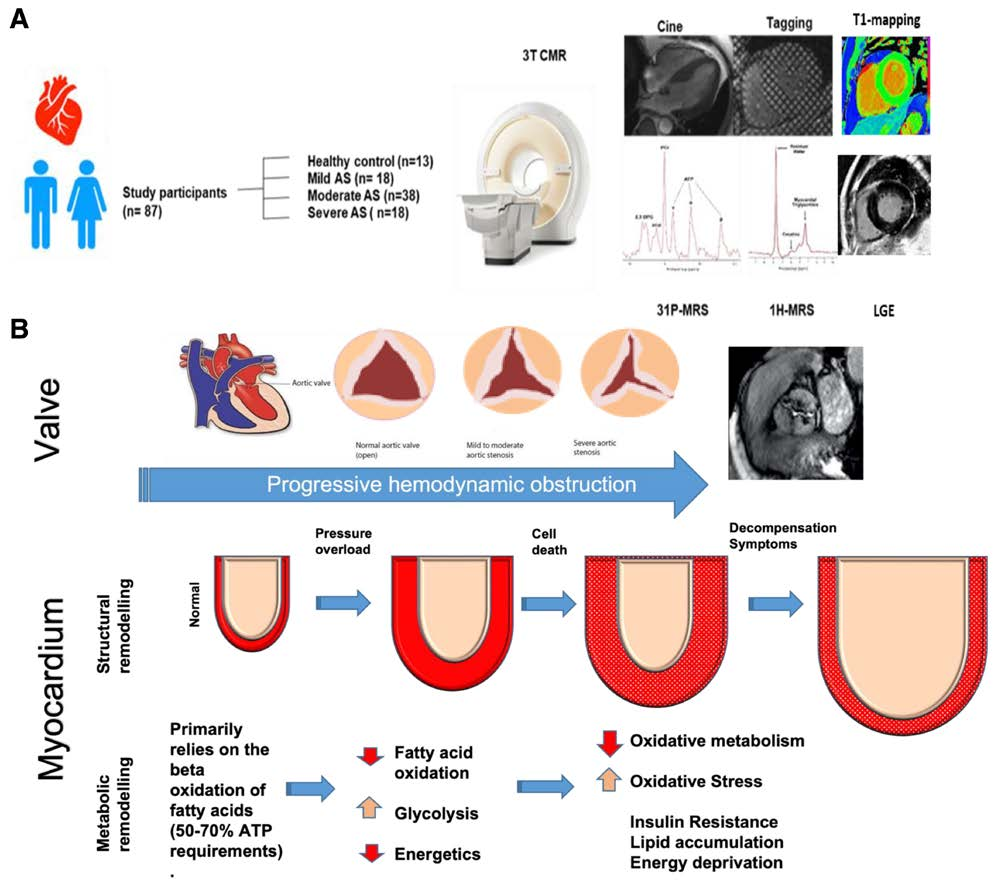
**Study design and rationale. A**, Schematic representation of study design showing the cardiac magnetic resonance (CMR) assessments used in the study including long-axis cine imaging, mid short-axis slice for tagging to assess left ventricular strain, T1 mapping and late gadolinium imaging, ^31^P-MRS spectrum with phosphocreatine and ATP peaks, ^1^H-MRS spectra with lipid and water peaks. **B**, Stages of structural and metabolic remodeling in aortic stenosis (AS) being explored in the study. 1H indicates proton spectroscopy; 31P, phosphorus spectroscopy; ATP, adenosine triphosphate; LGE, late gadolinium enhancement; and MRS, magnetic resonance spectroscopy.

It is not yet fully known why there is wide individual variation in the hypertrophic response to the same degree of pressure overload. Whether metabolic interactions play a causal role in disease progression remains unclear. The early metabolic change in pressure overload is a shift toward glucose use rather than fatty acids to produce adenosine triphosphate (ATP).^3^ This is believed to contribute to impaired cardiac energetics, which in animal studies precedes cardiac dysfunction and LV hypertrophy (LVH).^4^ Similar metabolic changes are also seen in established LVH, with a shift away from free fatty acid use toward glucose but also an uncoupling of glycolysis and glucose oxidation, again resulting in an altered energetic state.^5^

The myocardial phosphocreatine/ATP ratio, an index of cardiac bioenergetic state, is reduced in both animal and human models of myocardial hypertrophy^5^ and heart failure.^6^ It correlates not only with the degree of LVH and dysfunction but has also been shown to be a predictor of mortality.^7,8^ Thus, although both pressure overload and LVH are related to the metabolic changes seen in AS, their relative contributions are unknown. Given the direct coupling of ATP metabolism and contractile function, metabolic alterations are an attractive marker to identify individuals who are more vulnerable to LV failure in AS.

## METHODS

This was a prospective observational cohort study where 74 asymptomatic AS participants were recruited from outpatient valve clinics across 3 sites in the United Kingdom: Oxford University Hospital National Health Service Trust, Oxford, Great Western Hospital National Health Service Trust, Swindon, and Royal Berkshire Hospital, Reading. Thirteen healthy volunteers were recruited. All participants provided written informed consent. The study received approval from a Research Ethics Committee (South London, 18/LO/1981). The research was conducted in accordance with institutional procedures and the Declaration of Helsinki. The data that support the findings of this study are available from the corresponding author upon reasonable request.

### Study Design

Comprehensive baseline clinical characteristics and history were obtained using a standardized structured proforma and were completed from source clinic record data and patient questionnaires. AS severity for participation in the study was determined by outpatient echocardiographic findings fulfilling at least 2 criteria using a combination of peak velocity, aortic valve gradient, and estimated valve area as detailed below in the inclusion criteria.

#### Inclusion Criteria

Asymptomatic mild-to-severe AS with at least 2 criteria on routine echocardiogram—mild AS: peak velocity, Vmax<3 m/s; peak gradient, <30 mm Hg; mean gradient, <20 mm Hg; aortic valve area>1.5 cm^2^; moderate AS: Vmax<4 m/s; peak gradient, <60 mm Hg; mean gradient, <40 mm Hg; aortic valve area>1 cm^2^; severe AS: Vmax>4 m/s; peak gradient, >60 mm Hg; mean gradient, >40 mm Hg; aortic valve area<1 cm^2^, no other significant valvular pathology, no contraindication to MR imaging.

#### Exclusion Criteria

Known coronary artery disease (determined on a combination of clinical history, absence of symptoms, previous investigations including invasive or computed tomography coronary angiogram as well as absence of infarction on cardiac magnetic resonance [CMR]), presence of other underlying cardiomyopathy, LV ejection fraction <50%, uncontrolled hypertension, type 1 or type 2 diabetes, liver impairment, body mass index (BMI), >40 kg/m^2^, significantly impaired renal function (estimated glomerular filtration rate, <30 mL/min).

### Blood Sampling

Participants with AS underwent venous blood sampling for full blood count, renal and liver function, lipid profile, natriuretic peptide test, glucose, and free fatty acids. All participants were asked to fast 4 to 6 hours before the study visit.

### CMR and Late Gadolinium Enhancement Imaging

All participants were scanned in a fasted state (at least 6 hours fast) before study assessments. Cine images were acquired for cardiac volume analysis using a Siemens 3T Prisma MR system (Erlangen, Germany) using steady-state free precession cine imaging as previously described.^9^ Image analysis for atrial and ventricular volumes was performed offline using a semiautomated system (cmr42 Version 5.10.1; Circle Cardiovascular Imaging, Inc) in accordance with Society for Cardiovascular Magnetic Resonance guidelines.^10^

For LV wall thickness analysis, LV epicardial and endocardial contours were identified in end-diastole and end-systole, and planimetry was performed using semiautomated analysis software tools (cmr42 Version 5.10.1; Circle Cardiovascular Imaging, Inc). The maximum LV wall thickness was curated by an independent operator (SMonga; 4 years CMR experience) from the basal short-axis views of the left ventricle in end-diastole. Right and LV trabeculations were excluded from wall thickness measurements. Measurements were checked randomly by an independent operator (M.M.; 10 years, CMR experience).

Late gadolinium enhancement (LGE) was performed with an inversion-recovery–prepared, single shot gradient echo sequence after a 5- to 7-minute time delay subsequent to the administration of 0.3 mmol/kg of a gadolinium-based contrast agent (Dotarem). The inversion time was adjusted for optimal nulling of normal myocardium. LGE was analyzed qualitatively in terms of whether it was present or absent by 2 experienced CMR readers.

### Myocardial Tagging

Tagged MR images were acquired with an ECG-triggered segmented k-space fast gradient echo sequence with spatial modulation of magnetization in orthogonal planes,^11^ creating a square grid of parallel tag lines. A horizontal long-axis image and a short-axis image at the mid-ventricular level were acquired. Postprocessing analysis was performed using cardiac image modeller-Tag software (Auckland, New Zealand). From the mid short axis, peak systolic circumferential strain (CS) rate and diastolic CS rate were derived. From the horizontal long axis, peak systolic longitudinal strain rate and diastolic longitudinal strain rate were determined.

#### ^31^Phosphorus Magnetic Resonance Spectroscopy for Cardiac Energetics

Phosphorus magnetic resonance spectroscopy (MRS) was performed at rest on a 3T magnetic resonance imaging scanner (Magnetom Prisma; Siemens Healthineers). Participants were positioned supine with the center of dual-tuned ^1^H/^31^P surface coil (11 cm loop for ^31^P and butterfly for ^1^H, Rapid Biomedical, Rimpar, Germany) over their hearts in the isocentre of the magnetic resonance imaging system. We used single volume depth-resolved surface coil spectroscopy. This technique involves positioning of the voxel in the interventricular septum parallel to the receive coil, avoiding chest wall muscles. Three saturation bands are added to further suppress signals originating in chest wall and in the liver.^12^ At our center, standard operating procedures are followed for the positioning of the volume of interest, which removes any significant user variability in the acquisition of data. The results are then automatically generated from the acquired data using OXSA Matlab post processing tool^13^ (ie, a MATLAB implementation of the AMARES (Advanced Method for Accurate, Robust, and Efficient Spectral fitting) fitting routine by an experienced operator); thus, there is no significant interobserver variability. The fitted phosphocreatine and ATP signals were corrected for partial saturation, using literature values,^14^ before calculating the phosphocreatine/ATP ratio as phosphocreatine/average ATP or phosphocreatine/γ-ATP depending on spectral quality. The reported phosphocreatine/ATP was also corrected for blood signal contamination.^15^ While the variability of MRS is often cited as a limitation, current techniques typically yield a variability of 13% for phosphocreatine/ATP ratios, which is in the same range as LV volumes and functional assessment on CMR scanning and echocardiography.^16,17^

#### ^1^H-MRS for MTG Assessment

Myocardial ^1^H-MR spectra were obtained from the mid-interventricular septum as previously described.^18^ Spectroscopic acquisitions were performed using single-voxel water-suppressed stimulated echo acquisition mode spectroscopy sequence with ECG trigger at end-expiration to minimize motion artifacts following local SOPs. Twenty-five times water-suppressed spectra were acquired over 5 breath holds to measure myocardial triglyceride content (MTG) signal, and 3 times spectra without water suppression, acquired in an additional breath hold, thus 6 breath holds were involved in the acquisition of this data, with a total acquisition time of ≈10 minutes (giving the patients sufficient time to breath in between the breath holds). Spectra were analyzed using OXSA toolbox as previously described.^13^

MTG was calculated as a percentage relative to unsuppressed water: (signal amplitude of lipid/signal amplitude of water)×100. This technique has been shown previously to have an intrasubject coefficient of variation of 19% for lipid quantification with breath-holding at 3T.^18^

### Native T1 Mapping and ECV Estimation

Myocardial T1-mapping was performed using the ShMOLLI 5(1)1(1)1 sequence as previously published.^19^ Postcontrast T1-maps were acquired 15-minute post-administration of an intravenous gadolinium-based contrast agent (Dotarem). T1-maps were acquired in 1 mid-ventricular short-axis slice matched to cine image and images post-processed as previously described.^20^

R1 of blood and myocardium was calculated as R1=1/T1 for both native and postcontrast maps. Hematocrit was measured on a blood sample drawn on the same day, immediately before the CMR scan. Extracellular volume (ECV) was calculated using the equation:

ECV=(1−hematocrit)×(ΔR1 myocardium/ΔR1 blood)

Figure 1 gives a pictorial representation of the study design and protocol.

### Statistical Analysis

As the clinical grading of AS severity can often be discordant and does not follow a linear relationship with disease process, we chose to divide patients into quartiles of LV wall thickness (LVWT) and aortic valve gradient (AVG) to understand the relationship between myocardial metabolism and the disease process. LVWT was defined as the maximum wall thickness in the basal short-axis views of the LV in end-diastole. To investigate the relationship between myocardial metabolism and pressure overload, participants were divided into quartile groups based on peak AVG, derived from echocardiography, and LV pressure gradient (LVG, that is, aortic valve gradient, AVG+systolic blood pressure).

All normally distributed data (Kolmogorov-Smirnov test) are expressed as mean±SD. Non-normally distributed cardiac metabolism data is presented as median (interquartile range). Categorical data are presented as frequencies (%). Continuous variables were compared using a Student *t* test, Mann-Whitney *U* test, ANOVA with Bonferroni correction, or a Kruskal-Wallis test with Bonferroni correction. Ordered medians were compared for variables that were not normally distributed, that is, phosphocreatine/ATP and MTG content using the Jonckheere-Terpstra test. Categorical data were compared using Pearson χ^2^ test or Fisher exact test. Pearson and Spearman rank coefficients were determined to assess correlations. Correlation between categorical and continuous variables was performed using Kruskal-Wallis or Mann-Whitney *U* tests. Multivariable linear regression was performed using Spearman partial correlation analysis. A *P*<0.05 was considered significant. All statistical analyses were performed with IBM SPSS Statistics, Version 28 and GraphPad Prism (Version 9.0.2 for Windows; GraphPad Software, San Diego, CA).

## RESULTS

Seventy-four patients (70% men; mean age, 70±14 years; BMI, 27±4.5 kg/m^2^) with varying degrees of AS and 13 normal controls (54% men; mean age, 47±8 years; and BMI, 25±5 kg/m^2^) were studied.

### LVWT Quartiles

To assess the relationship between LVWT and metabolism, 87 participants were separated into quartiles based on LVWT.

#### Demographic and Clinical Characteristics

Participant demographic and clinical data is shown in Table S1. In the comparison of quartiles, Q1 were younger compared with others and with lower systolic blood pressure. All quartiles had higher number of men than women. All quartiles were matched in rest of the characteristics with no significant difference in blood glucose, total cholesterol, triglyceride and free fatty acid levels.

#### LV Structure and Function

LVWT (9.6 mm in Q1 versus 17.6 mm in Q4; *P*<0.001), LV mass index (50±12.5 kg/m^2^ in Q1 versus 81±13 kg/m^2^ in Q4; *P*<0.001) and LV end-diastolic volume (135±33 mL in Q1 versus 163±28 mL in Q4; *P*=0.04) showed a stepwise increase across the quartiles. Although LV ejection fraction was similar, and normal across the quartiles, peak systolic longitudinal strain (normal range −15% to −20%) was normal in Q1 (−15±0.97%) and impaired with increasing wall thickness (−10±1.8% in Q4; *P*<0.001). Peak systolic longitudinal strain correlated with LV mass index (*P*<0.001). Peak systolic CS despite reducing numerically across the quartiles (Q1, −18.6±1.8% versus Q4, −15.7±2.9%; *P*=0.03), overall remained in the normal range (−15% to −20%).^21^

**Figure 2.**
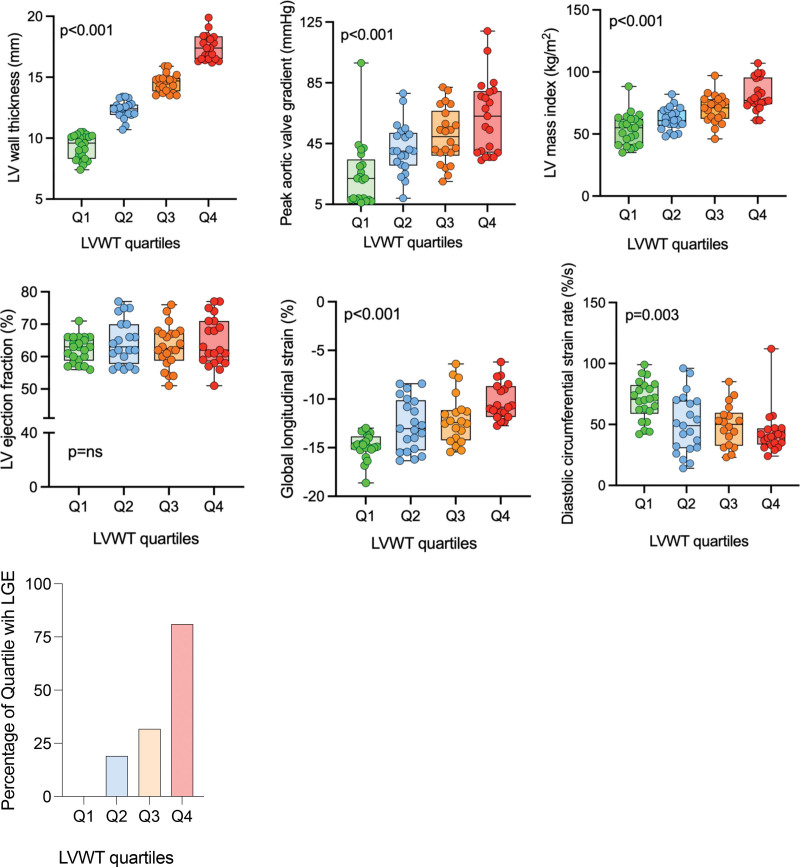
**Graphic representation of left ventricular structural parameters across the quartiles of left ventricular (LV) wall thickness (LVWT).** *P* values are from 1-way ANOVA with post hoc Bonferroni correction for all groups. LVWT quartiles are color coded; Quartiles: Q1, green; Q2, blue; Q3, orange; Q4, red. LGE indicates late gadolinium enhancement.

LV diastolic function indicated by early diastolic longitudinal strain rate (normal range, 41–36%/second)^22^ and early diastolic CS rate (normal range, 22–145%/second)^22^ showed a significant stepwise reduction across the increasing LVWT quartiles (Figure 2; Table S1).

When controlled for AV gradient, there was significant correlation between LVWT and peak systolic longitudinal strain (*r*=−0.53; *P*<0.001), CS (*r*=−0.37; *P*=0.003) and diastolic CS rate (*r*=−0.29; *P*=0.02, Table S2).

#### Myocardial Metabolism

**Figure 3.**
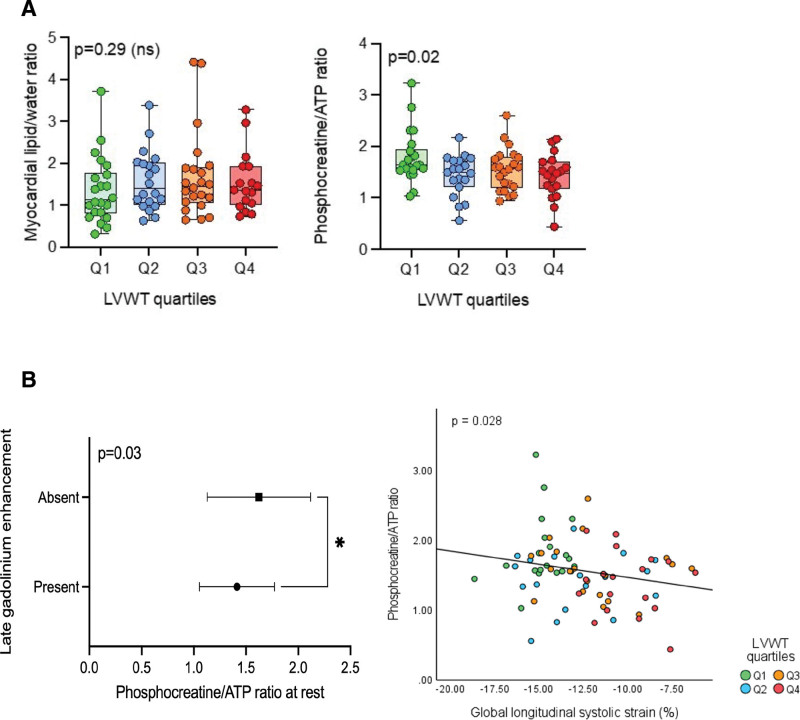
**Relationship between cardiac metabolism and left ventricular (LV) wall thickness (LVWT). A**, LV metabolic parameters across the quartiles of LVWT: *P* values for metabolic parameters are the result of ordered medians Jonckheere-Terpstra test across the groups from linear regression analysis. LVWT quartiles are color coded; Q1, green; Q2, blue; Q3, orange; Q4, red. **B**, Relationship between phosphocreatine (PCr)/ATP ratio and myocardial fibrosis on late gadolinium enhancement (LGE; **top**) and global longitudinal strain (**bottom**). **Top** plot represents mean with 95% CI values for PCr/ATP in the presence and absence of LGE. Presence or absence of LGE being a categorical variable. *P* value is derived from Spearman rank correlation analysis. **Bottom** plot shows Spearman correlation between PCr/ATP and global longitudinal strain in LVWT quartiles. LVWT quartiles are color coded; Q1, green; Q2, blue; Q3, orange; and Q4, red.

Myocardial phosphocreatine/ATP reduced by 10% in median across the increasing LVWT quartiles (ordered medians JT test; *P*=0.02). However, most of the reduction (9%) in the phosphocreatine/ATP occurred between Q1 and Q2 (Q1, 1.64 [1.53–1.94] versus Q2, 1.50 [1.21–1.71]; *P*=0.02; Figure 3A; Table S1). On partial correlation analysis, controlling for AV gradient, age, and BMI, there remained a significant relationship between LVWT and phosphocreatine/ATP (*r*=−0.20; *P*=0.04; Table S2).

MTG was numerically higher in Q2 (1.40) compared with Q1 (1.13) highlighting the early presence of steatosis with values remaining higher across Q2 to Q4 compared with Q1. Steatosis, however, did not progressively worsen with wall thickness across the quartiles (JT-ordered medians: *P*=0.29; Figure 3A; Table S1). On controlling for AGE, BMI, and AV gradient, MTG did have a significant association with LVWT (*r*=−0.27; *P*=0.03; Table S2).

On multivariate regression analysis with LVWT as dependent variable and LVG, MTG, BMI, age, and phosphocreatine/ATP as independent variables, only LV gradient (*r*=0.44; *P*≤0.001), BMI (*r*=0.26; *P*=0.006) and phosphocreatine/ATP (*r*=−0.20; *P*=0.03) were independent predictors of LV wall thickness. This highlights the importance of energetics in the hypertrophic process.

#### Myocardial Fibrosis on T1 Mapping, ECV Quantification, and LGE

##### T1 and ECV

Myocardial native T1 values increased progressively from Q2 to Q4 (Table S1, Q2, 1119±32 ms versus Q4, 1157±56 ms; *P*=0.29; normal values, 1096–1212 ms at 3T for our center). Similarly, % ECV increased progressively through the quartiles (Q1, 28±4.1% versus Q4, 30±7.9%; *P*<0.001), being abnormal in Q3 and Q4 (Table S1). Normal ECV range (27.5±3.5%).^23^

Presence of LGE was also seen to increase progressively across the quartiles, with the highest percentage of cases in Q4 (81% in Q4 versus 0% in Q1; *P*<0.001; Figure 2). Presence of LGE negatively correlated to cardiac energetics, phosphocreatine/ATP (*r*=−0.24; *P*=0.03). On controlling for LVG, age, and BMI, there was significant correlation between LVWT and presence of LGE (*r*=0.45; *P*<0.001), Table S2. There was no significant relationship between steatosis and myocardial fibrosis (*r*=0.22; *P*=0.05).

Thus, this analysis highlights the drop in energetics before a significant increase in LVWT. Phosphocreatine/ATP was an independent predictor of LVWT and had significant correlation with subclinical LV impairment and presence of myocardial fibrosis (Figure 3B).

### Pressure Overload Quartiles

To assess the relationship between pressure load and metabolism, all 87 participants were separated into quartiles based on peak AVG.

#### Demographic and Clinical Characteristics

Participants in Q1 were younger (mean age, 58±16 years) with lower average systolic BP (136±18 mm Hg). All quartiles were matched in rest of the characteristics (Table S3).

#### LV Structure and Function

**Figure 4.**
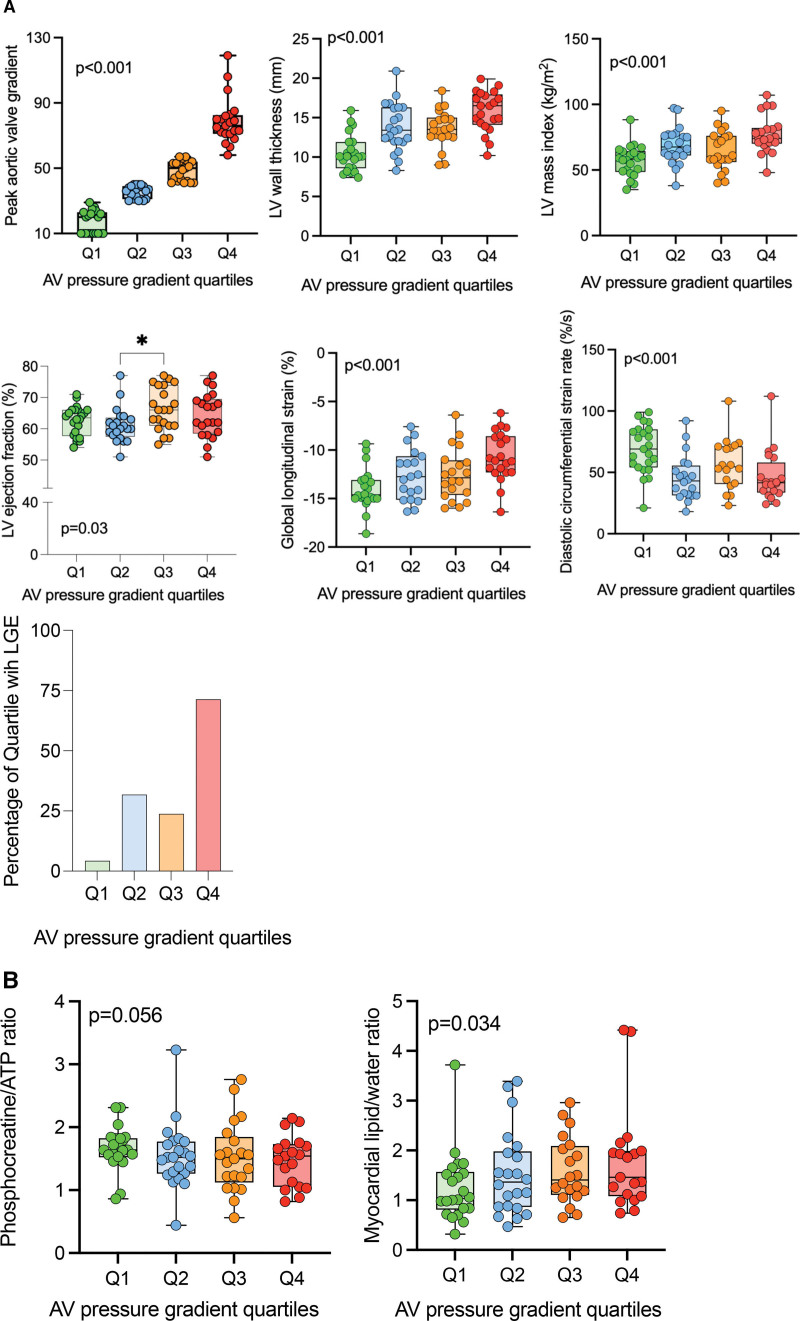
**Relationship between cardiac metabolism and aortic valve gradient (AVG). A**, Left ventricular structural parameters across the quartiles of AVG. **B**, Metabolic parameters across the quartiles of AVG. *P* values are from 1-way ANOVA with post hoc Bonferroni correction for all groups except for metabolic parameters where they are derived from ordered medians Jonckheere-Terpstra test from linear regression analysis. AVG quartiles are color coded; quartiles: Q1, green; Q2, blue; Q3, orange; Q4, red. AV indicates aortic valve; LGE, late gadolinium enhancement; and LV, left ventricle.

With increasing pressure load, there was an increase in LVWT (Q1, 10.4±2.3 versus 15.8±2.6 mm; *P*<0.001) and LV mass index. Longitudinal strain reduced across the increasing AVG quartiles (−13.8±2.2% in Q1 versus −10.6±2.5% in Q4; *P*<0.001). CS was lower in Q4 compared with others, and there was a step wise reduction in early diastolic CS rate (Q1, 69±19 versus Q4, 46±21%/s; *P*<0.001) across the ordered groups. On partial correlation controlling for LVWT, only CS had a significant association with AVG (*r*=−0.27; *P*=0.03; Figure 4A; Table S3).

#### Myocardial Metabolism

Myocardial phosphocreatine/ATP reduced across the increasing AVG quartiles (Q1, 1.64 [1.52–1.83] versus Q4, 1.51 [1.05–1.73], ordered medians JT test; *P*=0.056). In contrast, MTG increased significantly (by 42%) across the increasing AVG quartiles (JT-ordered medians, *P*=0.03; Figure 4B; Table S3). When controlled for LVWT, there was no significant relationship between AVG quartiles and phosphocreatine/ATP (*r*=−0.01; *P*=0.99) or MTG content (*r*=0.17; *P*=0.07; Table S4).

#### Myocardial Fibrosis on T1 Mapping, ECV Quantification, and Late Gadolinium Imaging

##### T1 and ECV

Native T1 and ECV were lower in Q2 to Q3 and then increased in Q4 (Table S3).

Areas of LGE were identified in overall 34% of patients with increasing prevalence across the quartiles, highest in Q4 (Figure 4A). The predominant pattern of LGE was patchy, mid-wall in the basal inferior and inferolateral regions. When controlled for LVWT, there was no significant relationship between presence of LGE and AVG alone (*r*=0.18; *P*=0.15; Table S4).

When assessed against total LV pressure load (AV gradient and systolic blood pressure), there was similar trend of increasing steatosis and reducing energetics across the quartiles, with total LV pressure significantly associated with steatosis (*P*=0.03). Further details available in the ^Supplemental Material^ (Tables S5 and S6; Figure S1).

### Clinical Grading of AS

Next, we divided patients into normal controls, mild-moderate AS, moderate AS, and severe AS groups based on their echocardiographic findings, we observed similar metabolic signature as seen on the quartiles. There was a significant reduction in cardiac energetics at mild-mod AS stage (*P*=0.03), and myocardial steatosis numerically increased across the groups (*P*=0.05). Details in Table S7 and Figure S6).

## DISCUSSION

Using the combination of advanced CMR and MRS, we show here for the first time that impaired energetics and myocardial steatosis precede a significant increase in LVWT and functional changes in AS.

### Effect of LV Hypertrophy

#### LV Structure/Function and LV Hypertrophy

Despite normal LV ejection fraction, there was convincing evidence of subclinical LV impairment with reduction in both systolic and diastolic strain parameters early, which worsened with disease progression. Impairments in longitudinal strain, CS, and CS rate were driven by increase in LVWT (*P*<0.001; *P*=0.003; *P*=0.02, respectively). Longitudinal strain was impaired earlier than CS in Q3 and Q4. This is in keeping with previous studies, which have demonstrated LVH to cause impaired longitudinal function in pressure overload despite normal ejection fraction. Circumferential and radial strain impairment occurs later and is also related to hypertrophy.^24^

#### Cardiac Metabolism and LV Wall Thickness

Previous studies have demonstrated that significant energetic impairment is already established in moderate-severe AS.^25^ We show here for the first time that energetic impairment is present even in milder forms of AS, more so, even in the absence of significant increase in LVWT (12.5 mm in Q2; Table S1). The metabolic changes described in this study, that is, reduced energetics and myocardial lipid accumulation support the metabolic substrate shift known to occur in AS—from fatty acids to glucose, subsequent increased glucose uptake and glycolysis with either no change or decrease in glucose oxidation. These adaptations improve myocardial oxygen efficiency but are associated with depletion of the energy reserve compound, phosphocreatine, while maintaining ATP levels until decompensation ensues.^26^ Reduced energetics levels could potentially be one of the driving factors in the development of LVH necessitating increased muscle mass to allow maintenance of normal contractile function in face of altered ATP production.

Further, we show here that the energetics worsen as LVWT increases and hypertrophy becomes established. Cardiac phosphocreatine/ATP was found to be an independent predictor of LVWT.

Similarly, myocardial steatosis is known to be well established in moderate-severe AS and related to LV systolic strain impairment.^27^ Despite this, whether a relationship between LVWT and MTG exists in AS remains unknown. We show here that presence of steatosis is seen earlier than moderate AS and before the development of significant hypertrophy. These findings support the hypothesis that metabolic changes as previously studied in animal and human models of AS^28,29^ occur prematurely and potentially drive or accelerate LVH and subsequent fibrosis and decompensation.

#### Myocardial Fibrosis and LVWT

Findings from the study are consistent with previous studies showing that replacement fibrosis seems to occur late and is irreversible.^30^ Presence of myocardial replacement fibrosis as seen on LGE was related significantly to LVWT (*P*<0.001). This is congruent with previous studies showing that development of hypertrophy is associated with myocyte apoptosis resulting in focal sites of replacement fibrosis, which may be important in the transition from compensated hypertrophy to heart failure.^31^ The novel finding from this study is that the presence of fibrosis is related to reduced phosphocreatine/ATP (*P*=0.03).

This would also explain findings from previous histological studies that show an association between myocardial fibrosis at the time of valve replacement and both impaired recovery of LV systolic function and poor long-term outcomes after valve replacement.^23^

Overall, this study demonstrates that energetic impairment precedes significant change in LVWT from baseline and potentially exacerbates the hypertrophic response once it has developed, with progression to subclinical contractile dysfunction and myocardial fibrosis.

Steatosis also is related to LVWT but does not seem to have a causal relationship with development of hypertrophy.

### Effect of Pressure Overload

#### LV Structure and Function and Pressure Overload

Both LVWT and LV mass index increased with increasing AV gradient quartiles, but this was secondary to AV gradient with no significant additional effect of systolic blood pressure (AVG and LVWT, *P*<0.001; LVG and LVWT, *P*=0.07 when controlled for AVG, LVG data presented in supplement). Myocardial strain was impaired despite normal EF but only CS impairment was related to pressure overload. This reiterates the findings from previous studies that LV strain impairment in AS is driven by geometric changes in LV secondary to hypertrophy than AV gradient itself.^32^

#### Cardiac Metabolism and Pressure Overload

MTG increased progressively across the pressure overload quartiles (both AV and LV gradients) with significant difference between the quartile groups. Total LV gradient had added impact on myocardial steatosis (*r*=0.24; *P*=0.03) than AV gradient alone (*P*=0.07), independent of LVWT. Thus, highlighting the benefit of blood pressure control in AS for preventing/reducing cardiac lipotoxicity.^33,34^ Phosphocreatine/ATP ratio reduced significantly across the increasing pressure overload quartiles, but this reduction did not correlate to the degree of pressure overload.

#### Myocardial Fibrosis and Pressure Overload

Myocardial fibrosis was present in all pressure overload quartiles and the percentage of cases increased across the quartiles. However, this was secondary to the increasing LVWT rather than the pressure gradient itself.

#### Limitations

This study is limited by a small sample size, in line with its proof-of-principle nature. Also, this is a cross-sectional study, hence not able to map the exact timing of metabolic changes in an individual’s disease course, but only predict that they are present at mild-moderate AS stage regardless of the duration of AS in a particular patient. Another limitation is that the control and AS groups were not age-matched and the implications of this will require further study. Also, when assessed as continuous variables the correlations shown in Figures S3 and S4 were on the lower end. Another limitation, LVWT measured was the maximal wall thickness in the LV basal segment. This was based on regression analysis where LVWT rather than mass/volume ratio was the significant variable. Future studies would be needed to fully understand the relationships explored in this study.

#### CONCLUSIONS

In this study, we show that myocardial energetic deficit occurs in mild-moderate AS and in the absence of clinically meaningful increase in LV wall thickness. We also show that myocardial steatosis occurs progressively with increasing severity of LV pressure loading. This suggests that metabolic changes precede any notable change in LVWT and may explain some of the variation in degree of LVH in AS. Whether cardiac metabolism can be altered in AS to modify the hypertrophic response is unknown but may be a potential therapeutic strategy in the future.

## ARTICLE INFORMATION

### Sources of Funding

Drs Monga, Mahmod, and Myerson have received research grant support from the British Heart Foundation for research into metabolic treatments in aortic stenosis (clinical research training fellowship number: FS/18/17/33514). Dr Myerson’s research is supported by the National Institute for Health Research (NIHR) Oxford Biomedical Research Center. Dr Valkovič is supported by a Sir Henry Dale Fellowship from the Wellcome Trust (No. 221805/Z/20/Z). Dr Rider is supported by a British Heart Foundation Senior Clinical Research Fellowship (FS/SCRF/22/32014). Drs Neubauer and Rider acknowledge support from the Oxford NIHR Biomedical Research Center and the British Heart Foundation Center of Research Excellence. Dr Valkovič also acknowledges the support from the Slovak grant agencies VEGA (Vedecká Grantová Agentúra; 2/0004/23) and APVV (Slovak Research and Development Agency; 21-0299).

### Disclosures

None.

### Supplemental Material

Tables S1–S7

Figures S1–S5

## Supplementary Material



## References

[1] Levy D Garrison RJ Savage DD Kannel WB Castelli WP. Prognostic implications of echocardiographically determined left ventricular mass in the Framingham heart study. N Engl J Med. 1990;322:1561–1566. doi: 10.1056/NEJM199005313222203 2139921 10.1056/NEJM199005313222203

[2] Frey N Katus HA Olson EN Hill JA. Hypertrophy of the heart: a new therapeutic target?. Circulation. 2004;109:1580–1589. doi: 10.1161/01.CIR.0000120390.68287.BB 15066961 10.1161/01.CIR.0000120390.68287.BB

[3] Lai L Leone T Keller M Martin OJ Broman AT Nigro J Kapoor K Koves TR Stevens R Ilkayeva OR. Energy metabolic reprogramming in the hypertrophied and early stage failing heart: a multisystems approach. Am Heart Assoc. 2014;7:1022–31. doi: 10.1161/CIRCHEARTFAILURE.114.001469 10.1161/CIRCHEARTFAILURE.114.001469 PMC4241130 25236884

[4] Hamirani YS Kundu BK Zhong M McBride A Li Y Davogustto GE Taegtmeyer H Bourque JM. Noninvasive detection of early metabolic left ventricular remodeling in systemic hypertension. Cardiology. 2016;133:157–162. doi: 10.1159/000441276 26594908 10.1159/000441276 PMC4677787

[5] Kolwicz SC Purohit S Tian R. Cardiac metabolism and its interactions with contraction, growth, and survival of cardiomyocytes. Circ Res. 2013;113:603–616. doi: 10.1161/CIRCRESAHA.113.302095 23948585 10.1161/CIRCRESAHA.113.302095 PMC3845521

[6] Ye Y Gong G Ochiai K Liu J Zhang J. High-energy phosphate metabolism and creatine kinase in failing hearts. Circulation. 2001;103:1570–1576. doi: 10.1161/01.cir.103.11.1570 11257087 10.1161/01.cir.103.11.1570

[7] Conway MA Allis J Ouwerkerk R Niioka T Rajagopalan B Radda GK. Detection of low phosphocreatine to ATP ratio in failing hypertrophied human myocardium by 31P magnetic resonance spectroscopy. Lancet. 1991;338:973–976. doi: 10.1016/0140-6736(91)91838-l 1681342 10.1016/0140-6736(91)91838-l

[8] Jameel M Zhang J. Myocardial energetics in left ventricular hypertrophy. Curr Cardiol Rev. 2009;5:243–250. doi: 10.2174/157340309788970379 20676284 10.2174/157340309788970379 PMC2822148

[9] Hudsmith LE Petersen SE Francis JM Robson MD Neubauer S. Normal human left and right ventricular and left atrial dimensions using steady state free precession magnetic resonance imaging. J Cardiovasc Magn Reson. 2005;7:775–782. doi: 10.1080/10976640500295516 16353438 10.1080/10976640500295516

[10] Schulz-Menger J Bluemke DA Bremerich J Flamm SD Fogel MA Friedrich MG Kim RJ von Knobelsdorff-Brenkenhoff F Kramer CM Pennell DJ. Standardized image interpretation and post processing in cardiovascular magnetic resonance: Society for Cardiovascular Magnetic Resonance (SCMR) board of trustees task force on standardized post processing. J Cardiovasc Magn Reson. 2013;15:35. doi: 10.1186/1532-429X-15-35 23634753 10.1186/1532-429X-15-35 PMC3695769

[11] Stuber M Spiegel MA Fischer SE Scheidegger MB Danias PG Pedersen EM Boesiger P. Single breath-hold slice-following CSPAMM myocardial tagging. Magn Reson Mater Phys Biol Med. 1999;9:85–91. doi: 10.1007/BF02634597 10.1007/BF02634597 10555178

[12] Bottomley PA Foster TB Darrow RD. Depth-resolved surface-coil spectroscopy (DRESS) for in Vivo 1H, 31P, and 13C NMR. J. Magn. Reson. 1984;59:338–342. doi: 10.1016/0022-2364(84)90179-3

[13] Purvis LAB Clarke WT Biasiolli L Valkovič L Robson MD Rodgers CT. OXSA: An open-source magnetic resonance spectroscopy analysis toolbox in MATLAB. PLoS One. 2017;12:e0185356. doi: 10.1371/journal.pone.0185356 28938003 10.1371/journal.pone.0185356 PMC5609763

[14] Tyler DJ Hudsmith LE Clarke K Neubauer S Robson MD. A comparison of cardiac ^31^ P MRS at 1.5 and 3 T. NMR Biomed. 2008;21:793–798. doi: 10.1002/nbm.1255 18512846 10.1002/nbm.1255

[15] Horn M Neubauer S Bomhard M Kadgien M Schnackerz K Ertl G. 31P-NMR spectroscopy of human blood and serum: first results from volunteers and patients with congestive heart failure, diabetes mellitus and hyperlipidaemia. MAGMA Magnetic Resonance Materials in Physics, Biology, and Medicine. 1993;1:55–60.

[16] Barbier P Mirea O Cefalù C Maltagliati A Savioli G Guglielmo M. Reliability and feasibility of longitudinal AFI global and segmental strain compared with 2D left ventricular volumes and ejection fraction: intra- and inter-operator, test–retest, and inter-cycle reproducibility. Eur Heart J Cardiovasc Imaging. 2015;16:642–652. doi: 10.1093/ehjci/jeu274 25564395 10.1093/ehjci/jeu274

[17] Tyler DJ Emmanuel Y Cochlin LE Hudsmith LE Holloway CJ Neubauer S Clarke K Robson MD. Reproducibility of 31 P cardiac magnetic resonance spectroscopy at 3 T. NMR Biomed. 2009;22:405–413. doi: 10.1002/nbm.1350 19023865 10.1002/nbm.1350

[18] Rial B Robson MD Neubauer S Schneider JE. Rapid quantification of myocardial lipid content in humans using single breath-hold 1 H MRS at 3 Tesla. Magn Reson Med. 2011;66:619–624. doi: 10.1002/mrm.23011 21721038 10.1002/mrm.23011 PMC3427889

[19] Piechnik SK Ferreira VM Dall’Armellina E Cochlin LE Greiser A Neubauer S Robson MD. Shortened Modified Look-Locker Inversion recovery (ShMOLLI) for clinical myocardial T1-mapping at 1.5 and 3 T within a 9 heartbeat breathhold. J Cardiovasc Magn Reson. 2010;12:69. doi: 10.1186/1532-429X-12-69 21092095 10.1186/1532-429X-12-69 PMC3001433

[20] Carapella V Puchta H Lukaschuk E Marini C Werys K Neubauer S Ferreira VM Piechnik SK. Standardized image post-processing of cardiovascular magnetic resonance T1-mapping reduces variability and improves accuracy and consistency in myocardial tissue characterization. Int J Cardiol. 2020;298:128–134. doi: 10.1016/j.ijcard.2019.08.058 31500864 10.1016/j.ijcard.2019.08.058

[21] Jeung M-Y Germain P Croisille P ghannudi SE Roy C Gangi A. Myocardial tagging with MR imaging: overview of normal and pathologic findings. Radiographics. 2012;32:1381–1398. doi: 10.1148/rg.325115098 22977026 10.1148/rg.325115098

[22] Moreira HT Nwabuo CC Armstrong AC Kishi S Gjesdal O Reis JP Schreiner PJ Liu K Lewis CE Sidney S. Reference ranges and regional patterns of left ventricular strain and strain rate using two-dimensional speckle-tracking echocardiography in a healthy middle-aged black and white population: the CARDIA study. J Am Soc Echocardiogr. 2017;30:647–658.e2. doi: 10.1016/j.echo.2017.03.010 28511859 10.1016/j.echo.2017.03.010 PMC5495603

[23] Puls M Beuthner BE Topci R Vogelgesang A Bleckmann A Sitte M Lange T Backhaus SJ Schuster A Seidler T. Impact of myocardial fibrosis on left ventricular remodelling, recovery, and outcome after transcatheter aortic valve implantation in different haemodynamic subtypes of severe aortic stenosis. Eur Heart J. 2020;41:1903–1914. doi: 10.1093/eurheartj/ehaa033 32049275 10.1093/eurheartj/ehaa033 PMC7242071

[24] Kosmala W Plaksej R Strotmann JM Weigel C Herrmann S Niemann M Mende H Störk S Angermann CE Wagner JA. Progression of left ventricular functional abnormalities in hypertensive patients with heart failure: an ultrasonic two-dimensional speckle tracking study. J Am Soc Echocardiogr. 2008;21:1309–1317. doi: 10.1016/j.echo.2008.10.006 19041574 10.1016/j.echo.2008.10.006

[25] Peterzan MA Clarke WT Lygate CA Lake HA Lau J Miller J Johnson E Rayner JJ Hundertmark MJ Sayeed RA. Cardiac energetics in patients with aortic stenosis and preserved versus reduced ejection fraction. Circulation. 2020;141:1971–1985. doi: 10.1161/CIRCULATIONAHA.119.043450 32438845 10.1161/CIRCULATIONAHA.119.043450 PMC7294745

[26] Peterzan MA Lygate CA Neubauer S Rider OJ. Metabolic remodeling in hypertrophied and failing myocardium: a review. American Journal of Physiology-Heart and Circulatory Physiology. 2017;313:H597–H616. doi: 10.1152/ajpheart.00731.2016 28646030 10.1152/ajpheart.00731.2016

[27] Mahmod M Bull S Suttie JJ Pal N Holloway C Dass S Myerson SG Schneider JE De Silva R Petrou M. Myocardial steatosis and left ventricular contractile dysfunction in patients with severe aortic stenosis. Circ Cardiovasc Imaging. 2013;6:808–816. doi: 10.1161/CIRCIMAGING.113.000559 23833283 10.1161/CIRCIMAGING.113.000559

[28] Chiu H-C Kovacs A Ford DA Hsu F-F Garcia R Herrero P Saffitz JE Schaffer JE. A novel mouse model of lipotoxic cardiomyopathy. J Clin Investig. 2001;107:813–822. doi: 10.1172/JCI10947 11285300 10.1172/JCI10947 PMC199569

[29] Marfella R Di Filippo C Portoghese M Barbieri M Ferraraccio F Siniscalchi M Cacciapuoti F Rossi F D’Amico M Paolisso G. Myocardial lipid accumulation in patients with pressure-overloaded heart and metabolic syndrome. J Lipid Res. 2009;50:2314–2323. doi: 10.1194/jlr.P900032-JLR200 19470430 10.1194/jlr.P900032-JLR200 PMC2759838

[30] Carabello BA. Aortic valve replacement should be operated on before symptom onset. Circulation. 2012;126:112–117. doi: 10.1161/CIRCULATIONAHA.111.079350 22753532 10.1161/CIRCULATIONAHA.111.079350

[31] Sabbah H. Apoptotic cell death in heart failure. Cardiovasc Res. 2000;45:704–712. doi: 10.1016/s0008-6363(99)00348-x 10728392 10.1016/s0008-6363(99)00348-x

[32] Cramariuc D Gerdts E Davidsen ES Segadal L Matre K. Myocardial deformation in aortic valve stenosis: relation to left ventricular geometry. Heart. 2010;96:106–112. doi: 10.1136/hrt.2009.172569 19710026 10.1136/hrt.2009.172569 PMC2802316

[33] Li J Kemp BA Howell NL Massey J Krzysztof B Nczuk M Huang Q Chordia MD Roy RJ Patrie JT. Metabolic changes in spontaneously hypertensive rat hearts precede cardiac dysfunction and left ventricular hypertrophy. J Am Heart Assoc. 2019;8:e010926. doi: 10.1161/JAHA.118.010926 30764689 10.1161/JAHA.118.010926 PMC6405673

[34] Polak-Iwaniuk A Harasim-Symbor E Gołaszewska K Chabowski A. How hypertension affects heart metabolism. Front Physiol. 2019;10:435. doi: 10.3389/fphys.2019.00435 31040794 10.3389/fphys.2019.00435 PMC6476990

