# Supplementary material for: Role of Cardiac Energetics in Aortic Stenosis Disease Progression: Identifying the High-risk Metabolic Phenotype
Source: Circ Cardiovasc Imaging. 2023 Oct 17;16(10):e014863. doi: 10.1161/CIRCIMAGING.122.014863 (PMC10581424; doi:10.1161/CIRCIMAGING.122.014863)
Supplement: Supplementary file 1 [file hci-16-e014863-s001.pdf]

Metabolic phenotyping in aortic stenosis: insights from a multi-parametric CMR study

SUPPLEMENTARY MATERIAL

LV Pressure Gradient quartiles analysis

87 participants were divided into quartiles based on total LV gradient (LVG = AV gradient + systolic blood pressure).

Demographic and clinical characteristics

Participants in Q1 were relatively younger (mean age 55 ± 13 years) with lower average systolic BP (124 ± 10 mmHg). All quartiles were matched in rest of the characteristics with no significant difference in blood glucose, total cholesterol, and triglyceride levels. Notably there was no difference in FFA levels across the quartiles. (**Table S5)**.

LV structure and function

As seen with AV gradient, with increasing LVG there was an increase in LVWT (Q1 10.1 ± 2.1 vs 15.3 ± 2.5 mm; p <0.001) and LVMI (63 ± 22 kg/m^2, Q1 vs 75 ± 16, Q4; p = 0.16). Although LVEF remained within normal limits in all the quartile groups**,** LS was impaired across the increasing LVG quartiles (-14.7 ± 1.4% in Q1 vs -10.6 ± 2.5% in Q4; p <0.001) along with stepwise reduction in early diastolic circumferential strain rate (Q1 66 ± 19 vs Q4 46 ± 22 100/s; p = 0.02; **Table S5 and Figure S5A).** When controlled for LVWT, there was no significant correlation between strain parameters and total LVG (**Table S6).**

Myocardial metabolism

Myocardial PCr/ATP was lower in Q2-Q4 compared to Q1. The reduction from Q1 to Q3 was statistically significant (Q1 1.64 vs Q3 1.42; p = 0.03) but the change across the quartiles was not statistically significant (ordered medians JT test, p=0.07). MTG

increased numerically (by 19%) across the increasing LVG quartiles (JT ordered medians p=0.08), with a 33% increase between Q1 and Q2 (Q1 1.08 [0.72, 1.73] vs Q2 1.44 [0.95, 1.81]; **Table S5** and **Figure S5B)**.

When controlled for LVWT variable, LVG correlated significantly only to MTG (r = 0.24, p = 0.03), not to PCr/ATP (p = 0.70). This relationship was not significant (r = 0.12, p = 0.14) when controlled for both LVWT and AV gradient (**Table S6).**

Myocardial Fibrosis on T1 mapping, ECV and LGE:

T1 and ECV: No significant trend was seen in myocardial T1 values and ECV across the LVG groups. Native T1 and ECV were low normal in Q1-3 and then increased in Q4 (1176 ± 74 ms, Q4 vs 1120 ± 29 ms, Q1; **Table S5**).

Percentage of LGE fibrosis was highest in Q4 (64% Q4 vs 5% Q1). No correlation was seen between LVG and presence of LGE (r = 0.06, p = 0.56; **Table S6).**

Thus, both the pressure gradient analysis indicate that the presence of myocardial steatosis is related to degree of pressure overload. These metabolic changes are predominantly driven by the AV gradient and not the total LV gradient.

Metabolic phenotyping in aortic stenosis: insights from a multi-parametric CMR study

S1

A.

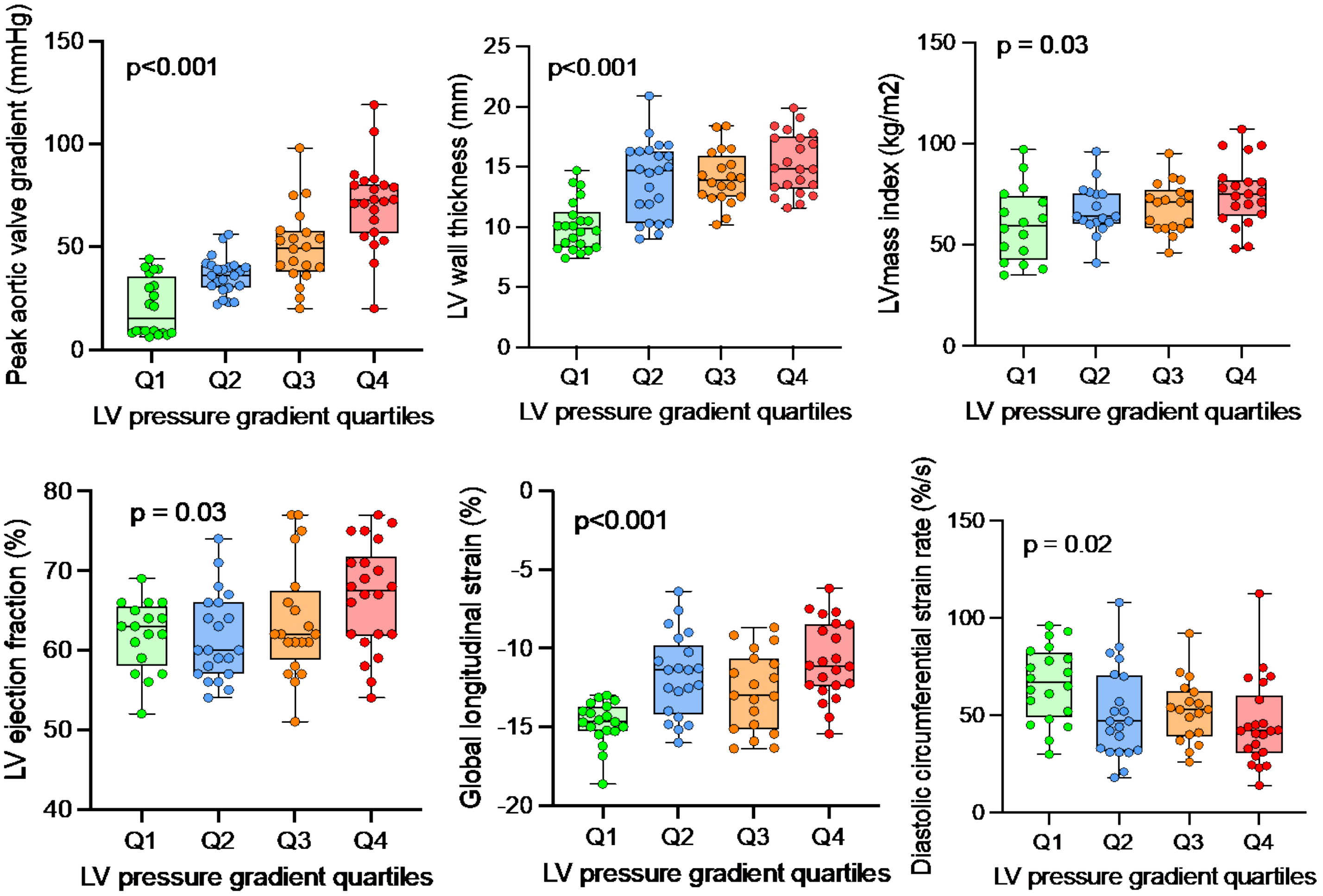

B

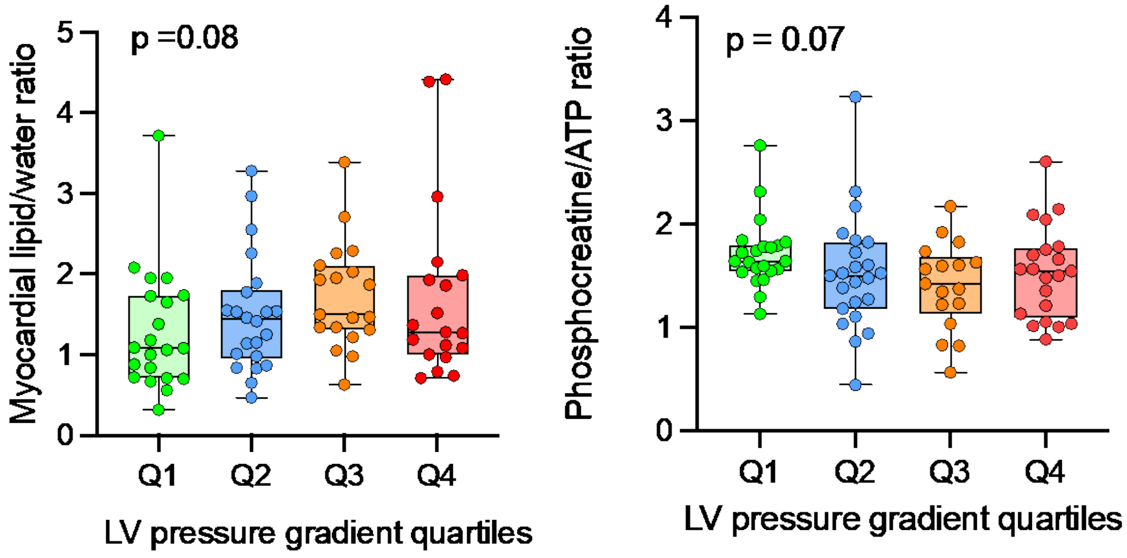

S2.

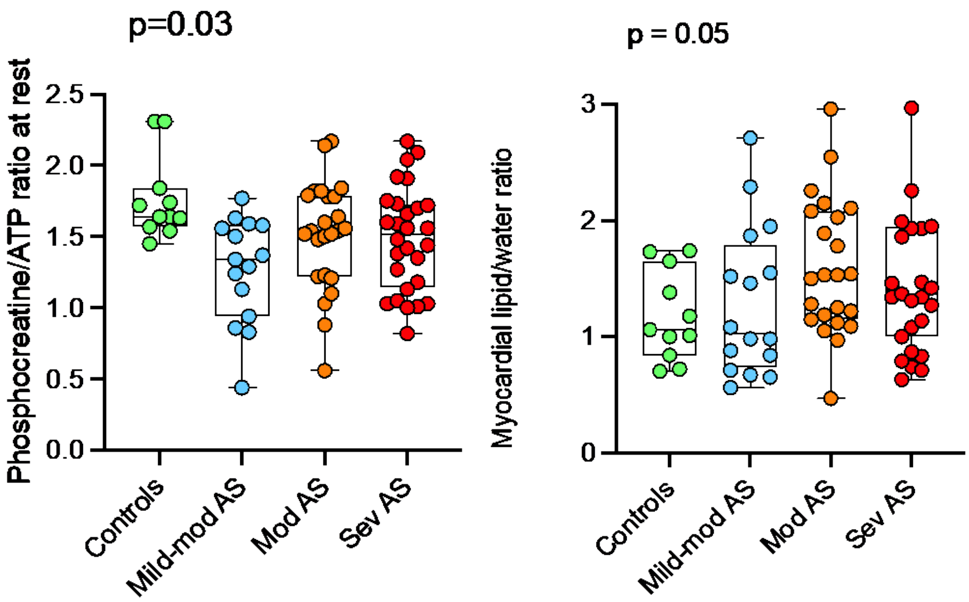
p=0.03
Phosphocreatine/ATP ratio at rest
2.5
2.0
1.5
1.0
0.5
0.0
Controls
Mild-mod AS
Mod AS
Sev AS
p = 0.05
Myocardial lipid/water ratio
3
2
1
0
Controls
Mild-mod AS
Mod AS
Sev AS

.

S3.

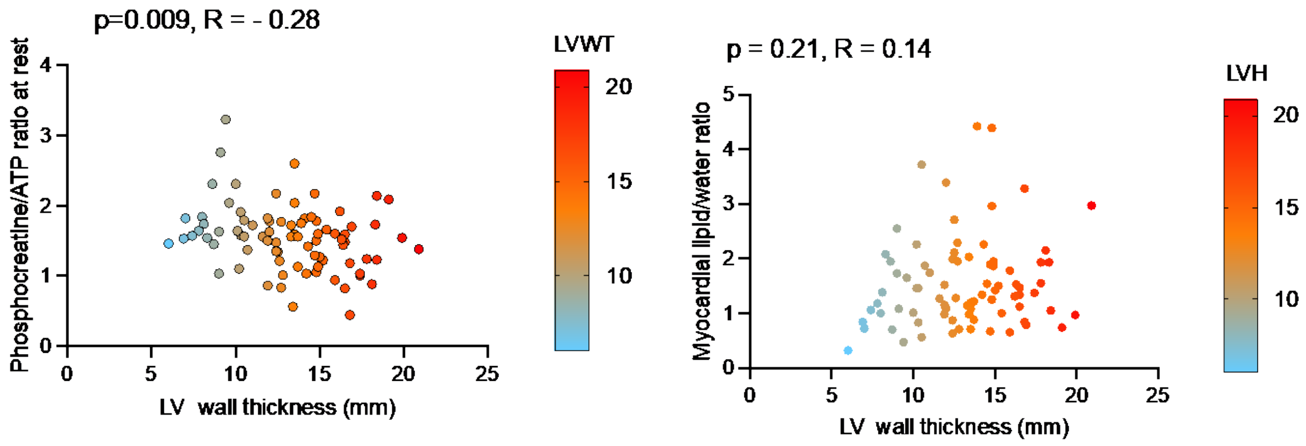
p=0.009, R = - 0.28
Phosphocreatine/ATP ratio at rest
LV wall thickness (mm)
LVWT
20
15
10
p = 0.21, R = 0.14
Myocardial lipid/water ratio
LV wall thickness (mm)
LVH
20
15
10

Metabolic phenotyping in aortic stenosis: insights from a multi-parametric CMR study

S4.

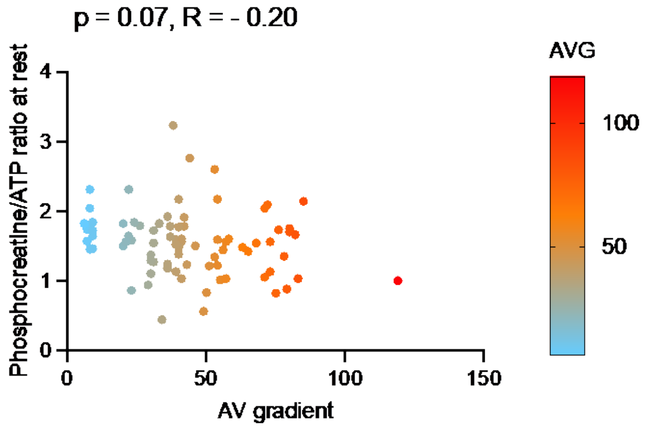

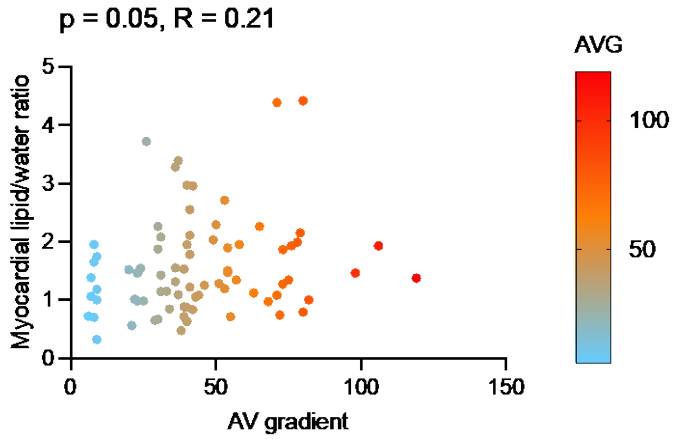

Metabolic phenotyping in aortic stenosis: insights from a multi-parametric CMR study

S5.

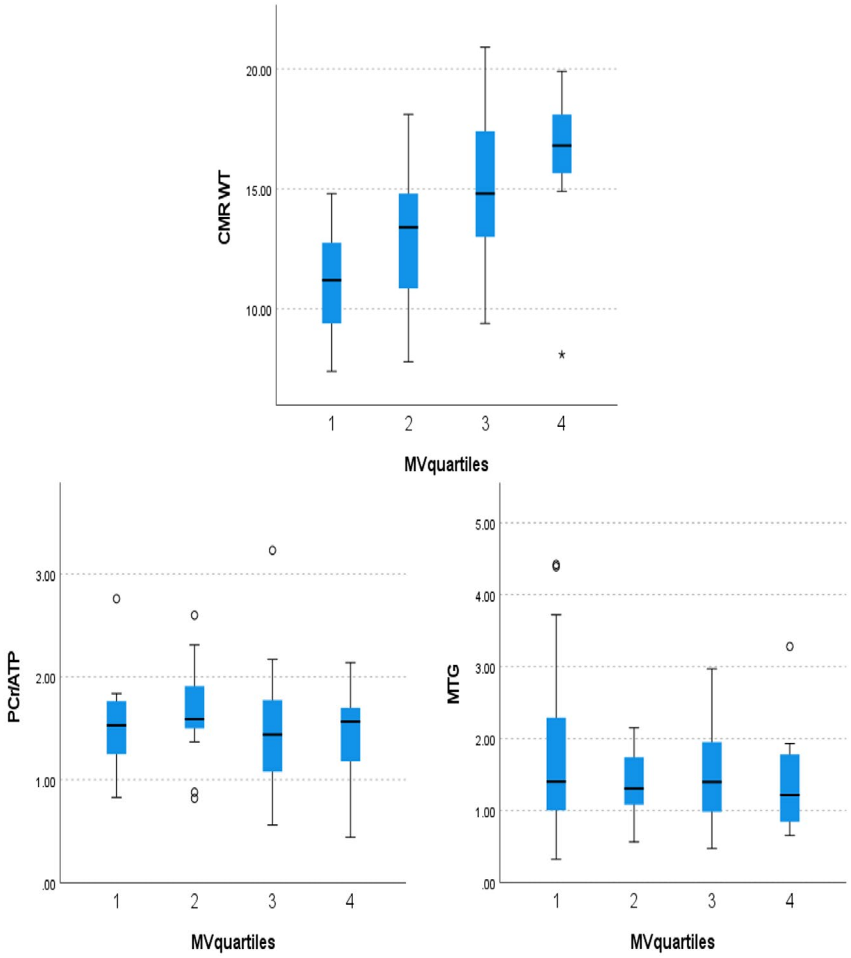

Supplementary Figure Legends

Figure S1: Left ventricular pressure gradient (LVG) quartiles

(A) Left ventricular structural parameters

(B) Metabolic parameters

P values are for 1-way ANOVA with post hoc Bonferroni correction for all groups except for metabolic parameters derived using ordered medians Jonckheere-Terpstra test from linear regression analysis. LVG quartiles are color coded; Q1, green; Q2, blue; Q3, orange; Q4, red.

Figure S2: Cardiac metabolic parameters across the normal controls and clinical grades of AS. P values are derived using ordered medians Jonckheere-Terpstra test from linear regression analysis for the study groups. P values and R values are from Spearman correlation analysis. Groups are color coded; Normals, green; Mild-mod AS, blue; Moderate AS, orange; Severe AS, red.

Figure S3: Correlation analysis: Cardiac metabolism and LV wall thickness (LVWT) as continuous variables across the whole study cohort.

P values are derived using ordered medians Jonckheere-Terpstra test from linear regression analysis for the study groups. P values and R values are from Spearman correlation analysis.

Figure S4: Correlation analysis: Cardiac metabolism and aortic valve gradient (AVG) as continuous variables across the whole study cohort.

P values are derived using ordered medians Jonckheere-Terpstra test from linear regression analysis for the study groups. P values and R values are from Spearman correlation analysis.

Figure S5: Mass/Volume (MV) quartiles analysis (p>0.05 for all analysis)

LV wall thickness (LVWT) increased across the MV quartiles, and cardiac energetics were reduced at a wall thicknes~14mm in the MV quartile data, but the trend was non-significant. In regression analysis, LVWT rather than mass/volume ratio was the significant variable.

Supplementary Tables

Table S1: **Demographics, clinical, biochemical characteristics and CMR indices of cardiac structure, function and metabolism in group based on LVWT.** Values are mean ± SD for continuous data unless stated otherwise and counts (percentages) for categorical data. P values are from ordinary one-way ANOVA for demographic, clinical and biochemical parameters. For cardiac structure and function, P values are for 1-way ANOVA with post hoc Bonferroni correction. *For cardiac metabolism, median and IQR are reported, and p values are the result of Jonckheere-Terpstra test across the groups from linear regression analysis (<0.05 significant in bold). LGE and medications were assessed as categorical variables.

For all tables, AS indicates aortic stenosis; BMI, body mass index; SBP, systolic blood pressure; DBP, Diastolic blood pressure; AF, atrial fibrillation; FFA, free fatty acids; TC, total cholesterol; TG, Triglycerides; BNP, brain natriuretic peptide; ARB, Angiotensin Receptor Blockers; ACE-I, Angiotensin Converting Enzyme Inhibitors.

AVG, aortic valve gradient; AVA, aortic valve area; LV, left ventricle; EDV, end-diastolic volume; ESV, end-systolic volume; SV, stroke volume; long, longitudinal; circ, circumferential; SR, strain rate; LA, left atrium; RV, right ventricle; LGE, late gadolinium enhancement; ECV, extracellular volume; MTG, myocardial triglyceride content; PCr/ATP, Phosphocreatine/ adenosine triphosphate ratio.

Table S2: Partial correlation analysis: LVWT and characteristics of cardiac structure, function and metabolism when controlled for aortic valve gradient (AVG) alone and AVG + Age + BMI (Body mass index). P <0.05 significant derived using Spearman's partial correlation analysis.

Table S3: Demographics, clinical, biochemical characteristics and CMR indices of cardiac structure, function and metabolism in group based on peak AV gradient.

Values are mean ± SD for continuous data unless stated otherwise and counts (percentages) for categorical data. P values are from ordinary one-way ANOVA for demographic, clinical and biochemical parameters. For cardiac structure and function, P values are for 1-way ANOVA with post hoc Bonferroni correction. *For cardiac metabolism, median and IQR are reported, and p values reported are the result of Jonckheere-Terpstra test across the groups from linear regression analysis (<0.05 significant in bold). LGE and medications were assessed as categorical variables.

Table S4: Partial correlation analysis: Aortic valve gradient and characteristics of cardiac structure, function and metabolism when controlled for left ventricular wall thickness. P < 0.05 significant, derived using Spearman's partial correlation method.

Table S5: Demographics, clinical, biochemical characteristics and CMR indices of cardiac structure, function and metabolism in group based on total LV gradient.

Values are mean ± SD for continuous data unless started otherwise and counts (percentages) for categorical data. P values are from ordinary one-way ANOVA for demographic, clinical and biochemical parameters. For cardiac structure and function, P values are for 1-way ANOVA with post hoc Bonferroni correction. *For cardiac metabolism, median and IQR are reported, and p values reported are the result of Jonckheere-Terpstra test across the groups from linear regression analysis (<0.05 significant in bold). LGE and medications were assessed as categorical variables.

Table S6: Correlation analysis: Total left ventricular gradient and characteristics of cardiac structure, function and metabolism when controlled for left ventricular wall thickness. P < 0.05 significant.

Table S7: **Demographics, clinical, biochemical characteristics and CMR indices of cardiac structure, function and metabolism in group based on clinical grading of AS.** Values are mean ± SD for continuous data unless stated otherwise and counts (percentages) for categorical data. P values are from ordinary one-way ANOVA for demographic, clinical and biochemical parameters. For cardiac structure and function, P values are for 1-way ANOVA with post hoc Bonferroni correction. *For cardiac metabolism, median and IQR are reported, and p values are the result of Jonckheere-Terpstra test across the groups from linear regression analysis (<0.05 significant in bold). LGE and medications were assessed as categorical variables.

Metabolic phenotyping in aortic stenosis: insights from a multi-parametric CMR study

Table S1

LVWT quartiles	Q1 (n=23)	Q2 (n=21)	Q3 (n=22)	Q4 (n=21)	P value
**LVWT (mm)**	9.6 ± 0.8	12.5 ± 0.5	14.5 ± 0.7	17.6 ± 1.3	**<0.001**
**Demographics data**					
Age (years)	53 ± 15	71 ± 10	69 ± 14	74 ± 11	***<0.001***
Male, n (%)	9 (39)	16 (76)	15 (68)	21(100)	***<0.001***
BMI (kg/m^2)	25 ± 5	27 ± 5	27 ± 4	28 ± 4	0.15
**Clinical data**					
SBP (mmHg)	126 ± 11	147 ± 16	139 ± 21	140 ± 14	***<0.001***
DBP (mmHg)	75 ± 12	72 ± 9	75 ± 8	72 ± 8	0.67
Heart rate (bpm)	63 ± 11	63 ± 11	66 ± 12	64 ± 9	0.80
Hypertension, n (%)	4 (17)	6 (29)	4 (18)	14 (66)	***<0.001***
AF, n (%)	1 (4)	1 (5)	4 (18)	2 (10)	0.36
**Biochemical data**					
Glucose (mmol/L)	5.0 ± 0.6	5.2 ± 0.5	5.3 ± 0.8	5.8 ± 1.5	0.12
FFA (mmol/L)	0.80 ± 0.5	0.55 ± 0.2	0.70 ± 0.3	0.62 ± 0.3	0.21
Butyrate (mmol/L)	0.15 ± 0.1	0.09 ± 0.0	0.14 ±0.1	0.15 ± 0.1	0.12
TC (mmol/L)	4.4 ± 0.8	4.6 ± 0.8	4.5 ± 0.9	4.4 ± 1.0	0.80
TG (mmol/L)	1.0 ± 0.4	0.96 ± 0.2	1.1 ± 0.5	0.93 ± 0.4	0.80
NT-pro BNP (ng/L)	140 ± 104	245 ± 327	442 ± 603	537 ± 868	0.24
**Medications**					
Beta blockers	3 (13)	1 (5)	1 (5)	4 (19)	0.44
ARB/ACE-I	4 (17)	6 (29)	4 (18)	14 (66)	0.004
Diuretics	0	1 (5)	2 (9)	3 (14)	0.81
Statins	3 (13)	11 (52)	6 (27)	10 (48)	0.26
**AV and LV parameters**					
AVG, mmHg	24 ± 22	42 ± 17	47 ± 16	69 ±25	**<0.001**
AVA, cm^2	2.5 ± 1.5	1.3 ± 0.5	1.0 ± 0.3	0.96 ± 0.3	**<0.001**
Peak velocity, m/sec	2.2 ± 0.98	3.2 ± 0.7	3.3 ± 0.6	3.9 ± 0.8	**<0.001**
LV EDV (ml)	135 ± 33	145 ± 31	157 ± 40	163 ± 28	**0.04**
LV ESV (ml)	51 ± 16	51 ± 14	59 ± 22	58 ± 17	0.30
LV SV (ml)	83 ± 18	95 ± 24	98 ± 22	101 ± 21	**0.04**
LV MI (kg/m^2)	50 ± 12.5	63 ± 9.6	73 ±11	81 ± 13	**<0.001**
LV EF (%)	63 ± 4	65 ± 7	63 ± 7	65 ± 8	0.57
Long strain (%)	-15 ± 0.97	-12.6 ± 2.9	-12.3 ± 2.6	-10 ± 1.8	**<0.001**
Circ strain (%)	-18.6 ± 1.8	-17 ± 4.3	-17.4 ± 3.7	-15.7 ± 2.9	**0.03**
Diastolic long SR (100/s)	26 ±13	28 ± 13	24 ± 8	21 ± 7	**0.02**
Diastolic circ SR (100/s)	69 ± 17	50 ± 24	48 ± 17	45 ± 19	**0.0007**
LGE present, n (%)	0	5 (24)	9 (40)	17 (81)	**<0.001**
ECV (%)	28 ± 4.1	27 ± 2.5	29 ± 7.2	30 ± 7.9	**<0.001**
Native T1 (ms)	1130 ± 28	1119 ± 32	1142 ± 83	1157 ± 56	0.28
**Other parameters**					
LA volume (ml)	60 ± 22	66 ± 19	100 ± 45	74 ± 28	**<0.001**
RV EF (%)	60 ± 5	62 ± 6	62 ± 6	61 ± 5	0.41

***Cardiac metabolism**					
MTG, %	1.13 (0.80, 1.78)	1.40 (1.0, 2.02)	1.46 (1.06, 1.91)	1.37 (1.01, 1.93)	0.29*
PCr/ATP	1.64 (1.53, 1.94)	1.50 (1.21, 1.71)	1.58 (1.19, 1.79)	1.48 (1.18, 1.70)	**0.02***

*P values for demographic, clinical and biochemical parameters were generated using ordinary one-way ANOVA comparison, and those for AV/LV and Other parameters were generated using one-way ANOVA comparison with post hoc Bonferroni correction. Medications has categorical variables; hence they were compared using t-tests and each quartile data detailed as counts (percentages) format. LGE was a categorical variable based on presence or absence, hence data were compared using t-tests and each quartile data detailed here in counts (percentages) format. *Cardiac metabolism data was not normally distributed; p values were generated by comparing ordered medians using Jonckheere-Terpstra test. Data for each quartile is, thus, represented as median (IQR). Significant (p<0.05) values are highlighted in bold.*

Table S2.

Relation with LVWT (Controlled for AVG)	r	P value
**LV function and fibrosis**		
Global long strain (%)	-0.53	**<0.001**
Global circ strain (%)	-0.37	**0.003**
Diastolic circ strain rate (100/s)	-0.29	**0.02**
Presence of LGE	0.43	**<0.001**
**Cardiac metabolism**		
MTG (lipid/water ratio)	-0.12	0.33
PCr/ATP	-0.29	**0.02**
**Controlled for AVG, Age & BMI**		
**LV function and fibrosis**		
Global long strain (%)	-0.45	**<0.001**
Presence of LGE	-0.44	**<0.001**
**Cardiac metabolism**		
MTG (lipid/water ratio)	-0.27	**0.03**
PCr/ATP	-0.20	**0.04**

P values derived from Spearman's partial correlation analysis, <0.05 significant highlighted in bold; r = correlation coefficient.

Metabolic phenotyping in aortic stenosis: insights from a multi-parametric CMR study

Table S3

AVG quartiles	Q1 (n=21)	Q2 (n=22)	Q3 (n=21)	Q4 (n=21)	P value
**AVG (mmHg)**	16 ± 8	36 ± 4	49 ± 6	79 ± 14	**<0.001**
**Demographics data**					
Age (years)	58 ± 16	68 ± 11	67 ± 17	74 ± 14	**0.005**
Male, n (%)	9 (39)	16 (76)	15 (68)	21(100)	**<0.001**
BMI (kg/m^2)	26 ± 5	27 ± 4	28 ± 5	26 ± 5	0.74
**Clinical data**					
Systolic BP (mmHg)	136 ± 18	135 ± 14	141 ± 20	141 ± 17	0.53
Diastolic BP (mmHg)	77 ± 11	72 ± 9	72 ± 8	72 ± 8	0.14
Heart rate (bpm)	65 ±12	64 ±11	63 ± 11	64 ± 9	0.95
Hypertension, n (%)	6 (23)	8 (36)	5 (24)	9 (43)	0.51
AF, n (%)	2 (1)	1 (4)	3 (14)	2 (10)	0.75
**Biochemical data**					
Blood glucose(mmol/L)	5 ± 0.5	5.1 ± 0.9	5.4 ± 0.4	5.5 ± 1.1	0.27
Free fatty acids (mmol/L)	0.59 ± 0.2	0.58 ± 0.2	0.65 ± 0.2	0.74 ± 0.4	0.46
Butyrate (mmol/L)	0.11 ± 0.0	0.14 ± 0.1	0.11 ± 0.1	0.15 ± 0.1	0.48
TC (mmol/L)	4.7 ± 0.9	4.3 ± 0.8	4.6 ± 0.7	4.3 ± 0.8	0.19
TG (mmol/L)	1.2 ± 0.5	0.99 ±0.4	1.1 ± 0.3	0.89 ± 0.4	0.19
NT-pro BNP (ng/L)	199 ± 252	221 ± 216	327 ± 578	608 ± 838	0.17
**Medications**					
Beta blockers	2 (1)	3 (14)	1 (5)	3 (14)	0.72
ARB/ACE-I	6 (23)	8 (36)	5 (24)	9 (43)	0.51
Diuretics	0	3 (14)	0	3 (14)	0.08
Statins	5 (22)	8 (36)	7 (33)	10 (48)	0.36
**AV and LV parameters**					
LVWT (mm)	10.4 ± 2.3	13.8 ± 3.0	13.6 ± 2.3	15.8 ± 2.6	**<0.001**
AVA, cm^2	2.6 ± 1.3	1.2 ± 0.23	1.0 ± 0.3	0.8 ± 0.2	**<0.001**
Peak velocity, m/sec	1.9 ± 0.5	2.9 ± 0.17	3.4 ± 0.2	4.3 ± 0.5	**<0.001**
LV EDV (ml)	139 ± 32	155 ± 39	146 ± 34	160 ± 30	0.21
LV ESV (ml)	53 ± 17	60 ± 22	49 ± 13	58 ± 18	0.13
LV SV (ml)	86 ± 17	94 ± 23	98 ± 27	98 ± 20	0.27
LV MI (kg/m^2)	53 ± 16	70 ± 14	64 ±15	77 ± 14	**0.009**
LV EF (%)	63 ± 5	61 ± 6	66 ± 7	64 ± 7	**0.034**
Long strain (%)	-13.8 ± 2.2	-12.6 ± 2.6	-12.5 ± 2.6	-10.6 ± 2.5	**<0.001**
Circ strain (%)	-17.4 ± 2.5	-15.7 ± 2.6	-17.3 ± 4.5	-16.8 ± 3.1	0.20
Diastolic long SR (100/s)	25 ± 12	29 ± 13	25 ± 8	21 ± 8	0.18
Diastolic circ SR (100/s)	69 ± 19	45 ± 18	57 ± 21	46 ± 21	**<0.001**
LGE present, n (%)	3 (13)	7 (32)	5 (24)	15 (71)	**<0.001**
ECV (%)	30 ± 5.8	28 ± 7.2	27 ± 4.9	29 ± 5.7	**<0.001**
Native T1 (ms)	1164 ± 54	1125 ± 40	1112 ± 71	1173 ± 16	**0.003**
**Other parameters**					
LA volume (ml)	67 ± 27	74 ± 43	80 ± 33	78 ± 29	0.63
RV ejection fraction (%)	60 ± 4	61 ± 7	63 ± 6	61 ± 5	0.60

***Cardiac metabolism**					
MTG (%)	1.03 (0.81, 1.56)	1.36 (0.86, 1.98)	1.40 (1.10, 2.09)	1.46 (1.08, 1.99)	**0.034***
PCr/ATP	1.64 (1.52, 1.83)	1.52 (1.26, 1.77)	1.50 (1.12, 1.84)	1.51 (1.05, 1.73)	0.056*

*P values for demographic, clinical and biochemical parameters were generated using ordinary one-way ANOVA comparison, and those for AV/LV and Other parameters were generated using one-way ANOVA comparison with post hoc Bonferroni correction. Medications has categorical variables; hence they were compared using t-tests and each quartile data detailed as counts (percentages) format. LGE was a categorical variable based on presence or absence, hence data were compared using t-tests and each quartile data detailed here in counts (percentages) format. *Cardiac metabolism data was not normally distributed; p values were generated by comparing ordered medians using Jonckheere-Terpstra test. Data for each quartile is, thus, represented as median (IQR). Significant (p<0.05) values are highlighted in bold.*

Table S4.

AVG Variables (Controlled for LVWT)	r	P value
**LV function and fibrosis**		
Global long strain (%)	-0.08	0.49
Global circ strain (%)	-0.27	**0.03**
Diastolic circumferential strain rate (100/s)	-0.05	0.69
Presence of LGE	0.18	0.15
**Cardiac metabolism**		
MTG (lipid/water ratio)	0.17	0.07
PCr/ATP	-0.01	0.99

P values derived from Spearman's partial correlation analysis, <0.05 significant highlighted in bold; r = correlation coefficient.

Metabolic phenotyping in aortic stenosis: insights from a multi-parametric CMR study

Table S5.

LVG quartiles	Q1 (n=22)	Q2 (n=23)	Q3 (n=20)	Q4 (n=22)	P value
Total LVG (mmHg)	142 ± 14	170 ± 6	193 ± 8	223 ± 18	**<0.001**
**Demographics data**					
Age (years)	55 ± 13	65 ± 17	70 ± 12	77 ± 10	**<0.001**
Male, n (%)	12 (55)	17 (74)	16 (80)	16(73)	0.30
BMI (kg/m^2)	25 ± 4	28 ± 4	26 ± 5	27 ± 5	0.42
**Clinical data**					
Systolic BP (mmHg)	124 ± 10	134 ± 11	143 ± 17	152 ± 18	**<0.001**
Diastolic BP (mmHg)	73 ± 12	74 ± 8	73 ± 9	74 ± 7	0.95
Heart rate (bpm)	64 ± 13	66 ± 10	60 ± 10	65 ± 9	0.21
Hypertension, n (%)	5 (23)	8 (35)	6 (30)	9 (41)	0.63
AF, n (%)	2 (9)	1 (4)	2 (10)	3 (14)	0.76
**Biochemical data**					
Blood glucose (mmol/L)	5.1 ± 0.6	5.1 ± 0.8	5.2 ± 0.5	5.5 ± 1.0	0.23
FFA (mmol/L)	0.53 ± 0.2	0.62 ± 0.2	0.78 ± 0.4	0.64 ± 0.2	0.18
TC (mmol/L)	4.5 ± 0.9	4.6 ± 0.7	4.4 ± 0.8	4.6 ± 0.9	0.85
TG (mmol/L)	1.1 ± 0.3	1.1 ± 0.4	0.98 ± 0.4	1.03 ± 0.6	0.94
NT-pro BNP (ng/L)	228 ± 281	313 ± 587	263 ± 432	521 ± 727	0.46
**Medications**					
Beta blockers	3 (14)	2 (9)	2 (10)	2 (9)	0.95
ARB/ACE-I	5 (23)	4 (18)	6 (30)	9 (41)	0.63
Diuretics	1 (4)	2 (9)	2 (10)	1 (5)	0.86
Statins	5 (23)	6 (26)	9 (45)	10 (45)	0.25
**AV and LV parameters**					
LVWT, mm	10.1 ± 2.1	13.9 ± 3.2	14.1 ± 2.3	15.3 ± 2.5	**<0.001**
AVA, cm^2	2.5 ± 1.3	1.3 ± 0.7	1.0 ± 0.3	0.9 ± 0.3	**<0.001**
Peak velocity, m/sec	2.1 ± 0.8	2.9 ± 0.4	3.4 ± 0.7	4.0 ± 0.6	**<0.001**
LV EDV (ml)	147 ± 45	144 ± 30	152 ± 26	156 ± 35	0.67
LV ESV (ml)	56 ± 24	55 ± 14	55 ± 13	53 ± 18	0.95
LV SV (ml)	91 ± 23	88 ± 21	98 ± 21	99 ± 22	0.27
LV mass index	63 ± 22	67 ± 13	69 ±12	75 ± 16	0.16
LV ejection fraction	62 ± 5	61 ± 5	64 ± 7	67 ± 7	**0.032**
Long strain (%)	-14.7 ± 1.4	-11.7 ± 2.6	-12.7 ± 2.5	-10.6 ± 2.5	**<0.001**
Circ strain (%)	-17.9 ± 2.4	-15.5 ± 3.1	-17.2 ± 3.3	-16.9 ± 3.6	0.11
Diastolic long SR (100/s)	25 ± 11	25 ± 11	29 ±11	21 ± 8	0.13
Diastolic circ SR (100/s)	66 ± 19	51 ± 24	52 ± 16	46 ± 22	**0.02**
LGE present, n (%)	1 (5)	10 (44)	5 (25)	14 (64)	**<0.001**
ECV (%)	28 ± 4.2	29 ± 8.3	28 ± 6.9	29 ± 3.3	0.81
Native T1(ms)	1120 ± 29	1126 ± 47	1120 ± 39	1176 ± 74	**0.003**
**Other parameters**					
LA volume (ml)	74 ± 45	68 ± 25	72 ± 26	86 ± 34	0.29
RV ejection fraction (%)	60 ± 5	60 ± 7	63 ± 6	63 ± 4	0.12

***Cardiac metabolism**					
MTG, %	1.08 (0.72, 1.73)	1.44 (0.95, 1.81)	1.50 (1.31, 2.11)	1.28 (1.00, 1.99)	0.08*
PCr/ATP	1.64 (1.54, 1.80)	1.50 (1.18, 1.82)	1.42 (1.13, 1.68)	1.54 (1.09, 1.77)	0.07*

*P values for demographic, clinical and biochemical parameters were generated using ordinary one-way ANOVA comparison, and those for AV/LV and Other parameters were generated using one-way ANOVA comparison with post hoc Bonferroni correction. Medications has categorical variables; hence they were compared using t-tests and each quartile data detailed as counts (percentages) format. LGE was a categorical variable based on presence or absence, hence data were compared using t-tests and each quartile data detailed here in counts (percentages) format. *Cardiac metabolism data was not normally distributed; p values were generated by comparing ordered medians using Jonckheere-Terpstra test. Data for each quartile is, thus, represented as median (IQR). Significant (p<0.05) values are highlighted in bold.*

Table S6.

**LVG Variables (Controlled for LVWT)**	**r**	**P value**
**LV function and fibrosis**		
Global long strain (%)	0.04	0.73
Global circ strain (%)	-0.19	0.13
Diastolic circ strain rate (100/s)	-0.13	0.29
Presence of LGE	0.07	0.56
**Cardiac metabolism**		
MTG (lipid/water ratio)	0.24	**0.03**
PCr/ATP	0.05	0.70

P values derived from Spearman’s partial correlation analysis, <0.05 significant highlighted in bold; r = correlation coefficient.

Metabolic phenotyping in aortic stenosis: insights from a multi-parametric CMR study

Table S7.

Groups	Normal (n=13)	Mild-mod AS (n=30)	Mod AS (n=22)	Sev AS (n=22)	P value
Age	51 ± 8	74 ± 8	66 ± 14	73 ± 13	<0.001
Male (%)	9 (69)	17 (56)	16 (73)	17 (77)	0.33
BMI	25 ± 4.7	26 ± 4.2	28 ± 3.6	26 ± 5.1	0.26
SBP	121 ± 8	137 ± 16	136 ± 18	145 ± 18	0.84
DBP	66 ± 5	73 ± 9	75 ±11	72 ± 16	0.78
HR	55 ± 7	63 ± 13	65 ± 17	60 ± 11	0.38
Glucose (mmol/L),	-	5.0 ± 0.2	5.4 ± 0.9	5.8 ± 1.5	0.16
Free fatty acids	-	0.53 ± 0.1	0.69 ± 0.3	0.66 ± 0.4	0.27
Butyrate levels	-	0.11 ± 0.0	0.13 ± 0.1	0.15 ± 0.1	0.14
TC (mmol/L),	-	4.6 ± 0.8	4.7 ± 0.9	4.4 ± 0.7	0.77
TG (mmol/L),	-	0.98 ± 0.3	1.1 ± 0.6	0.96 ± 0.2	0.91
NT-pro BNP (pg/ml)	-	248 ± 108	442 ± 388	584 ± 829	0.30
AF, %	0 (0)	2 (7)	3 (14)	1(4)	0.51
Hypertension, %	1 (9)	9 (30)	8 (36)	9 (41)	0.65
Medications					
Beta blockers		4 (13)	2 (9)	4 (18)	0.44
ARB/ACE-I		8 (27)	6 (27)	14 (66)	0.004
Diuretics		1 (5)	2 (9)	3 (14)	0.81
Statins		11 (52)	6 (27)	10 (48)	0.26
Peak AVG, mmHg	9.2 ± 4.3	31.8 ± 6.8	50.4 ± 7.3	81.6 ± 13.3	<0.001
LVWT, mm	9.0 ± 1.1	13.3 ± 2.8	13.8 ± 2.3	15.9 ± 2.7	<0.001
AVA, cm^2	3.7 ± 0.63	1.31 ± 0.26	1.16 ± 0.22	0.85 ± 0.19	<0.0001
Vmax (m/sec)	1.5 ± 0.29	2.6 ± 0.19	3.2 ± 0.19	4.1 ± 0.53	<0.0001
LVEDV (ml)	143 ± 32	156 ± 52	150 ± 31	156 ± 33	0.71
LV ESV (ml)	54 ± 18	65 ± 32	53 ± 15	55 ± 17	0.49
LV SV (ml)	90 ± 17	91 ± 22	97 ± 22	98 ± 23	0.67
LV MI (kg/m2)	59 ± 14	56 ± 11	68 ± 13	75 ± 15	0.0003
LV EF (%)	59 ± 14	60 ± 8	64 ± 6.5	67 ± 8	0.02
Systolic long strain (%)	-15 ± 1.7	-13 ± 2.4	-12 ± 2.8	-11 ± 2.4	0.003
Systolic circ strain (%)	-18 ± 2.4	-16 ± 2.5	-16 ± 4.1	-16 ± 4.0	0.53
Long SR (100/s)	33 ± 11	32 ± 9	27 ± 10	22 ± 8	0.003
Circ SR (100/s)	76 ± 14	51 ± 18	54 ± 22	47 ± 19	0.001
LA volume (ml)	61 ± 13	70 ± 33	71 ± 24	85 ± 30	0.08
RV EF (%)	60 ± 4	59 ± 6.2	61 ± 6	62 ± 5	0.30
PCr/ATP	1.77 ± 0.28	1.53 ± 0.47	1.49 ± 0.51	1.46 ± 0.43	0.03*
MTG (%)	1.17 ± 0.19	1.50 ± 0.87	1.66 ± 0.63	1.79 ± 1.11	0.05*

P values for demographic, clinical and biochemical parameters were generated using ordinary one-way ANOVA comparison, and those for AV/LV and Other parameters were generated using one-way ANOVA comparison with post hoc Bonferroni correction. Medications has categorical variables; hence they were compared using t-tests and each quartile data detailed as counts (percentages) format. LGE was a categorical variable based on presence or absence, hence data were compared using t-tests and each quartile data detailed here in

*counts (percentages) format. *Cardiac metabolism data was not normally distributed; p values were generated by comparing ordered medians using Jonckheere-Terpstra test. Data for each quartile is, thus, represented as median (IQR). Significant ($p<0.05$) values are highlighted in bold.*
